# Alveolar echinococcosis of the right adrenal gland: a case report and review of the literature

**DOI:** 10.1186/s13256-016-1115-0

**Published:** 2016-11-15

**Authors:** Silke Spahn, Birgit Helmchen, Urs Zingg

**Affiliations:** 1 Department of Surgery, Limmattal Hospital Zurich-Schlieren, Zurich, Switzerland; 2 Department of Pathology, Triemli Hospital, Zurich, Switzerland

**Keywords:** Alveolar echinococcosis, Multilocular echinococcosis, Adrenal gland, Incidentaloma, Cystic lesions

## Abstract

**Background:**

Extrahepatic manifestations of *Echinococcus multilocularis* are very rare, especially in the adrenal glands. To the best of our knowledge, only seven cases of adrenal alveolar echinococcosis have been reported, all from the Far East. All of these occurred exclusively in the right adrenal gland.

**Case presentation:**

We report a rare case of an extrahepatic alveolar echinococcosis in an asymptomatic 78-year-old white man with an incidentaloma of his right adrenal gland. After surgical resection and medical treatment with albendazole no recurrence of the disease appeared at 1-year follow-up.

**Conclusions:**

As the occurrence of *Echinococcus multilocularis* in Europe increases, alveolar echinococcosis should be included in the differential diagnosis of cystic adrenal incidentalomas.

## Background

An adrenal incidentaloma is an asymptomatic adrenal tumor that is incidentally discovered by an imaging test performed for other indications. Adrenal masses are the most common tumors in humans [1]. The prevalence is approximately 4.4 % in computed tomography (CT) scans [2]. Of all adrenal incidentalomas, 76 to 79 % are benign and non-functioning (adenoma 60 to 63 %, adrenal cyst 5 %, ganglioneuroma 3 to 5 %, myelolipoma 3 to 10 %, and adrenal hemorrhage 1 %) [2, 3]. In asymptomatic patients, Cushing’s syndrome (3 to 5 %) and pheochromocytoma (3 to 5 %) should be excluded by biochemical tests [2, 3]. The size of the tumor determines the prevalence of primary adrenal cortical carcinoma. Incidentalomas less than 4 cm are rarely malignant with a prevalence of 2 % [1]. Adrenocortical carcinomas are usually larger than 6 cm, are heterogenous and may be calcified [3]. Incidental adrenal carcinomas have a better prognosis than functioning or symptomatic adrenal carcinomas [2].

**Fig. 1:**
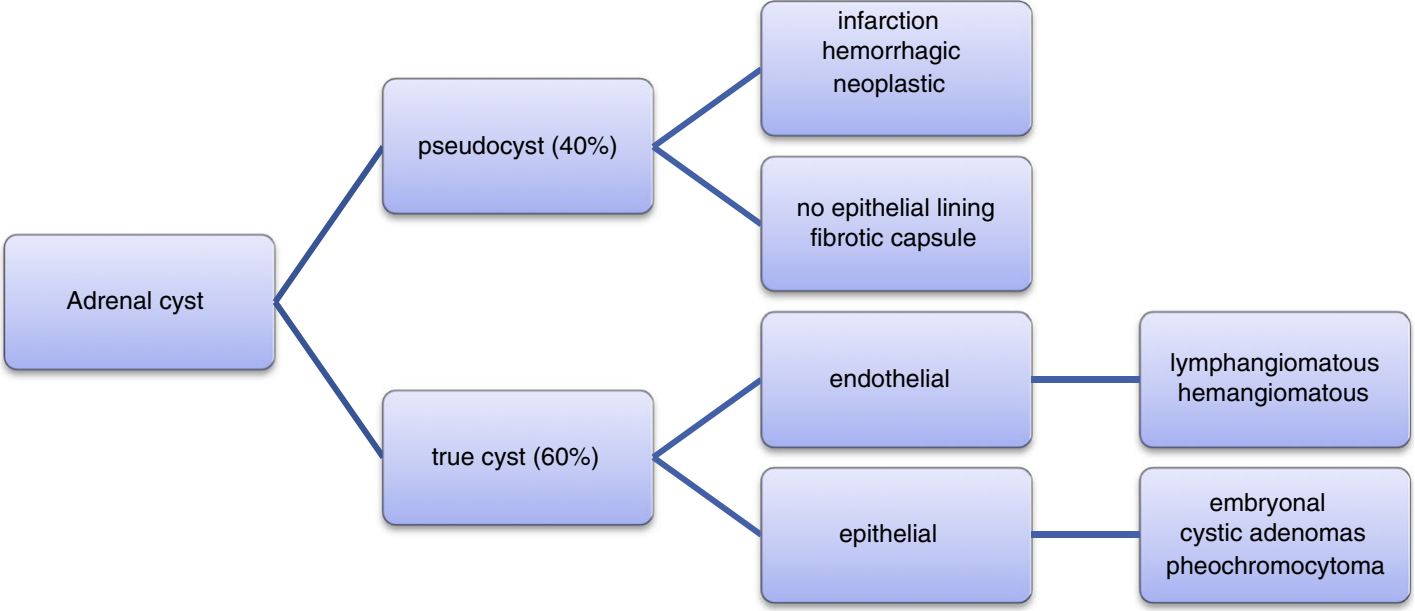
Differential diagnosis of adrenal cysts [4, 5]

Cystic adrenal lesions are uncommon with an incidence of 0.06 to 0.18 % at autopsy [3, 4]. Approximately 7 % of the adrenal cysts are hydatid cysts (*Echinococcus granulosus*) [5]. Infectious cysts usually show increased fluid density and calcifications in CT [3]. Cystic lesions also may be malignant or functional such as cystic adrenal carcinoma or cystic pheochromocytoma [3]. Figure 1 shows the differential diagnosis of adrenal cysts.

Surgical excision is indicated in hormonally active adrenal tumors or in case of suspicion of malignancy. As the prevalence of primary adrenal cortical carcinoma rises up to 25 % in patients with a tumor mass >6 cm adrenalectomy is indicated in these cases [1, 2]. Lesions between 4 and 6 cm are malignant in up to 6 % of cases. These lesions may be resected or must undergo close follow-up [1]. With the advance of laparoscopic surgery, some authors recommend surgery for smaller lesions as morbidity of the procedures is usually low [6].

Echinococcosis is a zoonotic infection of humans caused by *Echinococcus* tapeworms. The more prevalent cystic echinococcosis (hydatidosis) is caused by *Echinococcus granulosus*. Years after ingestion of the eggs, hydatids are found in the liver or lungs and rarely in other organs. Alveolar echinococcosis is confined to the northern hemisphere, in particular to North America/Canada, Central Europe, Russia, Western China and Japan [4, 7–9]. The fox tapeworm *Echinococcus multilocularis* is the parasite responsible for alveolar echinococcosis. The eggs are excreted with fox feces and accidentally orally ingested by humans as aberrant hosts. The embryos (oncospheres) hatch from the eggs and penetrate the intestinal wall. They are distributed via the blood stream or lymphatic system to settle in different organs. Approximately 99 % of cases of alveolar echinococcosis are located in the liver [7]. It takes 5 to 15 years until the first symptoms such as abdominal pain or hepatomegaly may appear [8]. The diagnosis is often incidental by ultrasound or CT. Serological tests have had a sensitivity of 94 to 100 % and a specificity of 95 to 96 % [8, 10]. In the early stages the lesions are small and cystic; later, additional fibrotic and/or calcified capsules may be seen [8].

An alveolar echinococcosis may be misdiagnosed as a malignant tumor as the multilocular alveolar cysts grow infiltratively in organs.

## Case presentation

**Fig. 2:**
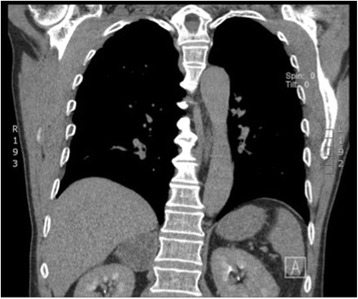
Computed tomography image demonstrates incidentaloma of 6 cm in the right adrenal gland

**Fig. 3:**
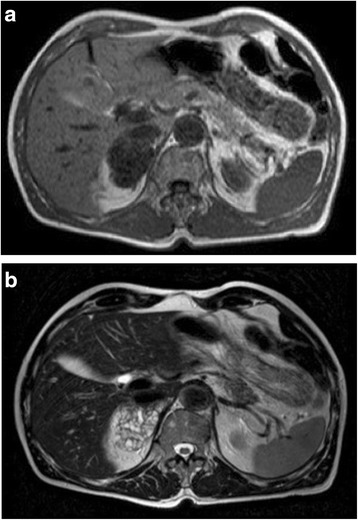
**a**, **b** Magnetic resonance imaging T1-weighted and T2-weighted images of right adrenal incidentaloma

**Fig. 4:**
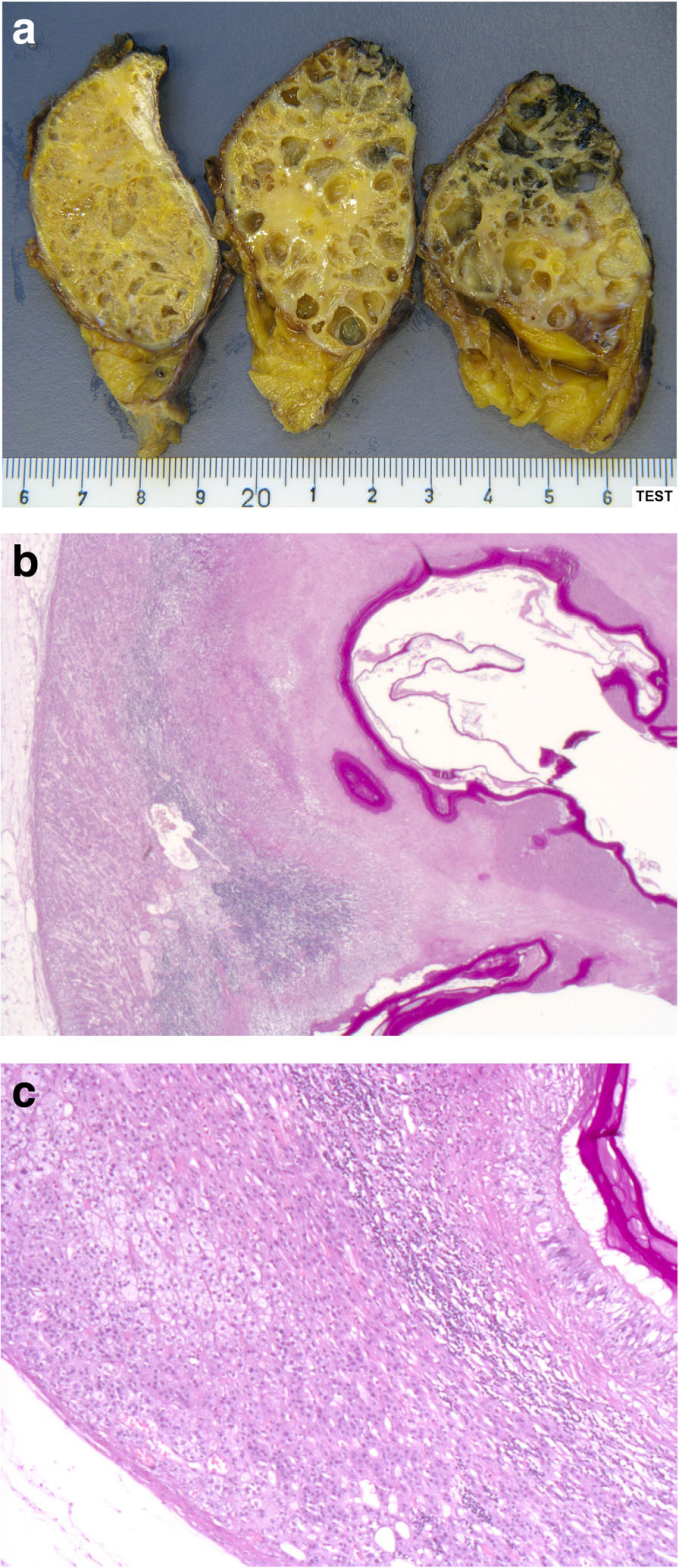
**a**–**c** Macroscopic and microscopic aspects of the resected specimen

**Fig. 5:**
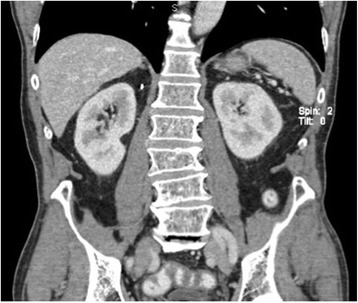
Follow-up computed tomography scan

A 78-year-old white man with productive cough as his single symptom presented to the pneumological unit in our hospital. The clinical findings including blood tests (complete blood cell count, electrolytes, serum biochemistry profile) were normal. CT scans of his thorax demonstrated, in addition to a chronic bronchitis, a large incidentaloma (6 cm) of his right adrenal gland (Fig. 2). Further imaging with magnetic resonance demonstrated a multilocular cystic mass with hypointensity in T1-weighted images and hyperintensity in T2-weighted images (Fig. 3). After injection of contrast the cystic walls enhanced and an infiltration to the liver was suspected. All hormonal tests illustrated a normal function of his adrenal glands; Cushing’s syndrome could be excluded by normal salivary cortisol at midnight (6.7 nmol/l), hyperaldosteronism by normal plasma aldosterone renin ratio (5.7 ng/mU) and pheochromocytoma by normal 24-hour urine catecholamines and metanephrines. In suspicion of a cystic malignant tumor of his right adrenal gland a diagnostic laparoscopy and open adrenalectomy were performed. On intraoperative examination, the incidentaloma infiltrated his inferior vena cava and segment VI of his liver. The macroscopic aspect was different from a classic malignant tumor and a benign etiology was suspected. Due to this intraoperative evaluation and the advanced age of the patient a limited resection without reconstruction of his inferior vena cava was performed. The resected mass was 7.2×7.4×3.5 cm and consisted of multiloculated cysts with scolices, necrosis, and inflammation (Fig. 4). The pathological results reported alveolar echinococcosis of his right adrenal gland. This result was confirmed by serological tests: *Echinococcus granulosus* hydatid fluid (EgHF)-enzyme-linked immunosorbent assay (ELISA), EgP-ELISA, AgB-EITB Western blot, Em18-ELISA, and Em2G11-ELISA. A lifelong treatment with albendazole was installed postoperatively. Follow-up after 1 year with clinical examination as well as CT scans showed no recurrence (Fig. 5).

## Discussion

Alveolar adrenal echinococcosis is rare and has been reported in seven cases so far, all from the Far East (Table 1). Similar to our patient, physical examination, complete blood cell count, biochemical tests, hormonal examinations, and urine analysis were normal in all cases.

**Table 1:** Cases of alveolar adrenal echinococcosis in the literature

Author	Case	Localization	Size	Age	Sex
Maru *et al*., 2007 [12]	1	Right adrenal gland	5.5×3 cm	79	M
Kamishima *et al*., 2009 [4]	2	Right adrenal gland	6×3 cm	77	M
Ran *et al*., 2012 [9]	3	Right adrenal gland	5×10×5 cm	45	M
	4	Right adrenal gland	5×12×6 cm	56	F
Chu *et al*., 2013 [13]	5	Right adrenal gland	8×7×6 cm	28	M
Huang and Zheng, 2013 [7]	6	Right adrenal gland	5.8×9.5 cm	45	M
	7	Right adrenal gland	5×11.2 cm	56	F

*F* female, *M* male

Imaging tests demonstrated in almost all cases an infiltration into the inferior vena cava, the liver, or the right renal vessel. Three of them were incidentalomas. Radiological findings and intraoperative findings suggested a malignant tumor in six cases and a benign cyst in one case. Furthermore, all reported adrenal lesions of alveolar echinococcosis were larger than 5 cm at diagnosis so that an adrenalectomy was indicated. In all cases, the diagnosis of alveolar echinococcosis in the adrenal gland was made postoperatively by the pathologist.

All reported cases including ours were located in the right adrenal gland. It is unclear how the parasites reach this organ. After ingestion of the parasitic eggs the oncospheres pass the intestinal wall and enter the blood or lymphatic system. The most probable way of infecting the right adrenal gland would be an invasion of the larvae continuously from the liver. Dissemination through the blood or lymphatic system directly to the adrenal gland is theoretically possible as well.

Despite being rare, preoperative diagnosis of echinococcosis is an advantage as these patients need to undergo a preoperative medical treatment [11]. Therefore, we suggest that patients with cystic lesions of the adrenal gland, especially the right adrenal gland, should undergo serological testing of different types of echinococcosis. For the planning of the surgical procedure in patients with cystic adrenal masses, the surgeon needs to think of a possible echinococcosis, and an extension of the surgery including liver resection and vascular reconstruction may be warranted.

## Conclusions

As alveolar echinococcosis is an increasing disease in Europe and extrahepatic manifestations such as in the adrenal glands are possible, *Echinococcus multilocularis* should be included in the differential diagnosis of cystic adrenal incidentalomas.

Patients with an infiltrative cystic incidentaloma of the right adrenal gland should undergo a serological test of alveolar echinococcosis. Confirmed alveolar echinococcosis mandates a radical adrenalectomy with possible extension to liver resection and vascular reconstruction and a preoperative and postoperative treatment with albendazole.

## Abbreviations

CT: Computed tomography
ELISA: Enzyme-linked immunosorbent assay

## References

[1] Fassnacht M, Arlt W, Bancos I, Dralle H, Newell-Price J, Sahdev A, Tabarin A, Terzolo M, Tsagarakis S, Dekkers OM. Management of adrenal incidentalomas: European Society of Endocrinology Clinical Practice Guideline in collaboration with the European Network for the Study of Adrenal Tumors. Eur J Endocrinol. 2016;175(2):G1–34. 10.1530/EJE-16-0467 27390021

[2] O’Neill CJ, Spence A, Logan B, Suliburk JW, Soon PS, Learoyd DL, Sidhu SB, Sywak MS. Adrenal incidentalomas: risk of adrenocortical carcinoma and clinical outcomes. J Surg Oncol. 2010;102(5):450–3. 10.1002/jso.21553 20734420

[3] Bittner 4th JG, Brunt LM. Evaluation and management of adrenal incidentaloma. J Surg Oncol. 2012;106(5):557–64. 10.1002/jso.23161 22623268

[4] Kamishima T, Harabayashi T, Ishikawa S, Kubota KC, Nonomura K, Omatsu T, Onodera Y, Shirato H, Terae S. Alveolar hydatid disease of the adrenal gland: computed tomography and magnetic resonance imaging findings. Jpn J Radiol. 2009;27(5):225–8. 10.1007/s11604-009-0326-3 19554417

[5] Otal P, Escourrou G, Mazerolles C, Janne d’Othee B, Mazghani S, Musso S, Colombier D, Rousseau H, Joffre F. Imaging features of uncommon adrenal masses with histopathologic correlation. Radiographics. 1999;19(3):569–81. 10.1148/radiographics.19.3.g99ma07569 10336189

[6] Muth A, Hammerstedt L, Hellström M, Sigurjonsdottir HA, Almqvist E, Wängberg B. Cohort study of patients with adrenal lesions discovered incidentally. Br J Surg. 2011;98(10):1383–91. 10.1002/bjs.7566 21618498

[7] Huang M, Zheng H. Primary alveolar echinococcosis (*Echinococcus multilocularis*) of the adrenal gland: report of two cases. Int J Infect Dis. 2013;17(8):653–5. 10.1016/j.ijid.2013.03.011 23769454

[8] Knapp J, Sako Y, Grenouillet F, Bresson-Hadni S, Richou C, Gbaguidi-Haore H. Comparison of the serological tests ICT and ELISA for the diagnosis of alveolar echinococcosis in France. Parasite. 2014;21:34. 10.1051/parasite/2014037 PMC4111071 25058754

[9] Ran B, Tuergan A, Shao YM, Jiang TM, Li HT, Wang YJ, Wen H. Alveolar echinococcosis of the adrenal gland: brief review of two cases. Chin Med J (Engl). 2012;125(9):1656–7. 22800838

[10] Pektas B, Altintas M, Akpolat N, Gottstein B. Evaluation of the diagnostic value of the ELISA tests developed by using EgHF, Em2 and Emll/3-10 antigens in the serological diagnosis of alveolar echinococcosis. Mikrobiyol Bul. 2014;48(3):461–8. 10.5578/mb.7742 25052112

[11] Arif SH, Shams-Ul-Bari, Wani NA, Zargar SA, Wani MA, Tabassum R, Hussain Z, Baba AA, Lone RA. Albendazole as an adjuvant to the standard surgical management of hydatid cyst liver. Int J Surg. 2008;6(6):448–51. 10.1016/j.ijsu.2008.08.003 18819855

[12] Maru S, Yamashita N, Shinno Y. Adrenal multilocular echinococcosis: a case report. Japanese J Urol. 2007;98(4):643–5. 10.5980/jpnjurol1989.98.643 17564109

[13] Chu ZG, Lv FJ, Zhu ZY, Ouyang Y. Extrahepatic primary adrenal alveolar echinococcosis: a review. Surg Infect (Larchmt). 2013;14(4):418–21. 10.1089/sur.2012.037 23859690

